# High activity and low toxicity of a novel CD71-targeting nanotherapeutic named The-0504 on preclinical models of several human aggressive tumors

**DOI:** 10.1186/s13046-021-01851-8

**Published:** 2021-02-10

**Authors:** Elisabetta Falvo, Verena Damiani, Giamaica Conti, Federico Boschi, Katia Messana, Patrizio Giacomini, Michele Milella, Vincenzo De Laurenzi, Veronica Morea, Gianluca Sala, Giulio Fracasso, Pierpaolo Ceci

**Affiliations:** 1 CNR – National Research Council of Italy, Institute of Molecular Biology and Pathology, Rome, Italy; 2 Center for Advanced Studies and Technology (CAST), Department of Medical Oral and Biotechnological Sciences, University of Chieti-Pescara, Chieti, Italy; 3 Department of Neurological and Movement Sciences, University of Verona, Verona, Italy; 4 Department of Computer Science, University of Verona, Verona, Italy; 5 IRCCS Regina Elena National Cancer Institute, Oncogenomics and Epigenetics, Rome, Italy; 6 Oncologia Medica, Azienda Ospedaliera Universitaria Integrata (AOUI), Verona, Italy; 7 Department of Medicine, University of Verona, Verona, Italy; 8 Thena Biotech, Latina, Italy

**Keywords:** Tumor targeted therapy, Preclinical studies, Human ferritin, Transferrin receptor 1 (CD71), Breast cancer, Gastrointestinal cancer

## Abstract

**Background:**

Ferritin receptor (CD71) is an example of a very attractive cancer target, since it is highly expressed in virtually all tumor types, including metastatic loci. However, this target can be considered to be inaccessible to conventional target therapies, due to its presence in many healthy tissues. Here, we describe the preclinical evaluation of a tumor proteases-activatable human ferritin (HFt)-based drug carrier (The-0504) that is able to selectively deliver the wide-spectrum topoisomerase I inhibitor Genz-644282 to CD71-expressing tumors, preventing the limiting toxic effects associated with CD71-targeting therapies.

**Methods:**

CD71 expression was evaluated using flow cytometry and immunohistochemistry techniques. The-0504 antiproliferative activity towards several cancer cell lines was assessed in vitro. The-0504 antitumor efficacy and survival benefit were evaluated in different human tumors, which had been grown either as xenografts or patient-derived xenografts in mice. The-0504 toxicology profile was investigated in multiple-cycle repeat-dose study in rodents.

**Results:**

In vitro studies indicate that The-0504 is highly specific for CD71 expressing cells, and that there is a relationship between CD71 levels and The-0504 anticancer activity. In vivo treatments with The-0504 showed a remarkable efficacy, eradicating several human tumors of very diverse and aggressive histotypes, such as pancreas, liver and colorectal carcinomas, and triple-negative breast cancer.

**Conclusions:**

Durable disease-free survival, persistent antitumor responses after discontinuation of treatment and favorable toxicology profile make The-0504 an ideal candidate for clinical development as a novel, CD71-targeted, low-toxicity alternative to chemotherapy.

**Supplementary Information:**

The online version contains supplementary material available at 10.1186/s13046-021-01851-8.

## Background

Triple-negative breast (TNBC) and gastrointestinal (GI) cancers are among the deadliest forms of cancer. TBNC is a subtype of breast cancer that is estrogens receptor negative, progesterone receptor negative and human epidermal growth factor receptor 2 (HER2) negative based on immunohistochemistry. TNBC is characterized by its unique molecular profile, aggressive nature, distinct metastatic patterns and lack of targeted as well as standard treatment options [1–3]. GI cancer includes cancers of the anus, colon and rectum (colorectal cancers), esophagus and stomach (gastroesophageal cancers), liver, gallbladder, pancreas and small intestine. Collectively, it represents one of the greatest public health issues in Europe and, indeed, worldwide, leading to million global deaths [4–6]. For both GI and TNBC patients there is an urgent need to develop novel therapies to improve outcomes.

In this paper we report the preclinical evaluation of a novel nanotherapeutic agent named The-0504. This product is a protein-drug complex, based on a modified version of human ferritin heavy chain (HFt). HFt-based nanotherapeutics have been recently attracting growing interest in the field of cancer drug delivery, due to their excellent biocompatibility, selectivity for cancer over normal cells, binding to a large number of different human tumors and ability to encapsulate in their internal cavity high amounts (20–120 mol/mol) of drugs belonging to different classes [7–22]. HFt is effectively taken up and rapidly internalized by virtually all types of cancer cells via the transferrin receptor 1 (TfR1, CD71) [23–26]. CD71 is highly expressed by virtually all tumor types, including metastatic loci, since rapidly growing tumor cells need large amounts of iron. Unsurprisingly, CD71 expression in gastrointestinal and breast tumors inversely correlates with survival [27, 28].

**Fig. 1:**
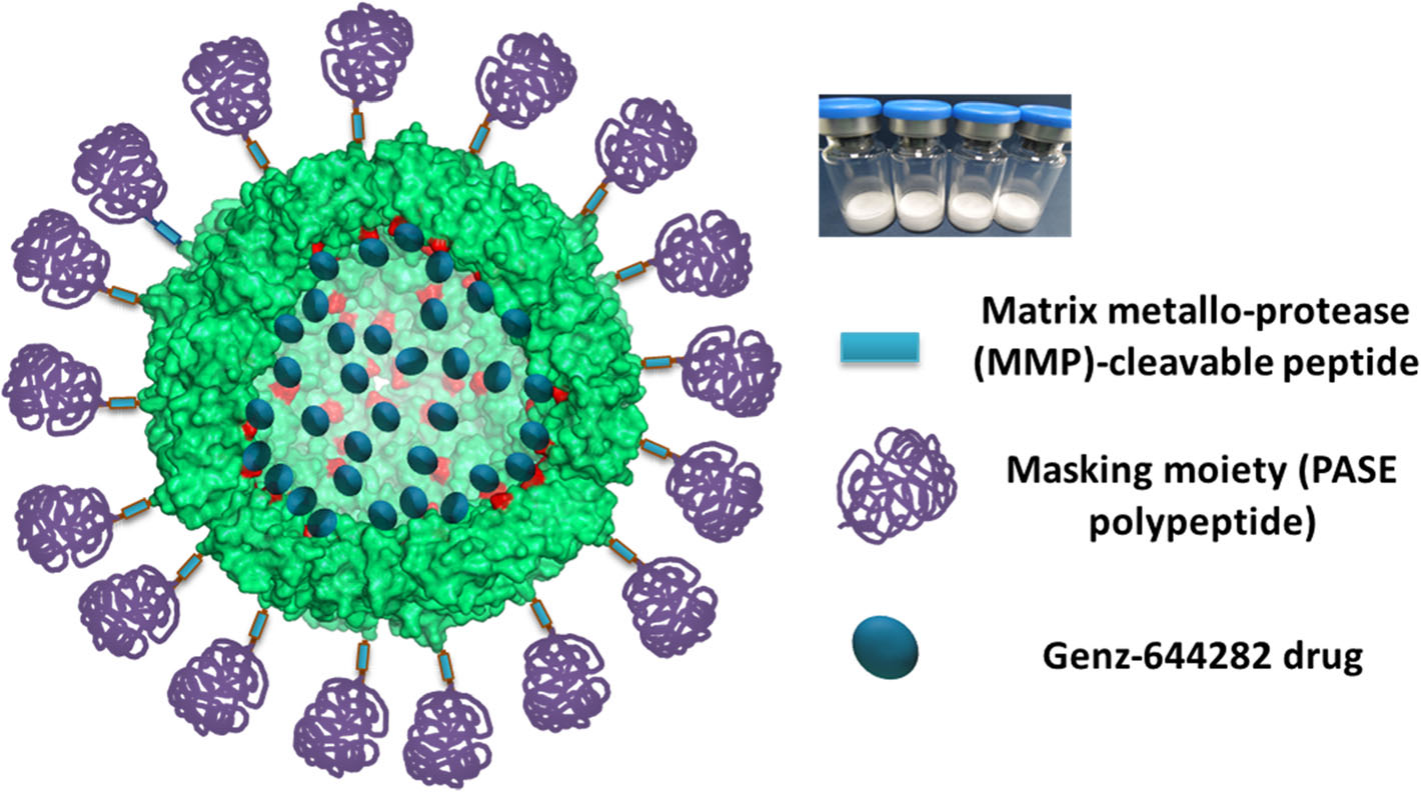
Schematic representation of The-0504. Metalloprotease cleavable sequence and masking polypeptides (named PASE) are colored light blue and purple, respectively. Genz-644282 drug is colored blue. All other protein residues are light green. To allow the internal surface of the protein to be visualized (lighter colors), only 18 monomers of 24 are shown. The picture has been generated with PyMol and GNU Image Manipulation Program

To increase wild-type HFt selectivity for cancer cells over healthy ones, we have previously developed a masked HFt variants, named HFt-MP-PAS/E, which exploits the unique conditions of the tumor microenvironment to fulfill its function (Fig. 1). HFt-MP-PAS/E variants are inactive during circulation and in normal tissues and is only activated by specific matrix-metalloproteases (MMP 2/9) that are expressed in the tumor microenvironment [29, 30]. The masking moiety present on the protein surface both extends protein half-life in the bloodstream as compared to native HFt [29–32], and increases protein chances to recirculate until it binds at the highly CD71 dense, but dimensionally small, tumor site.

A nanotherapeutic named The-0504 was obtained by incorporating the Genz-644282 drug in the internal cavity of a novel variant, named The-05, characterized by the masking moiety PASE. The details of The-05 and The-0504 production and characterization are reported in a recently published paper [33]. Genz-644282 is a non-camptothecin topoisomerase I inhibitor, with half maximal inhibitory concentrations (IC50s) ranging from 1.8 nM to 1.8 μM, which is active against a large number of human tumor cell lines [34], including camptothecin-resistant cell lines [35].

In the present work, the antitumor activity of The-0504 was investigated both in vitro, on ten different cancer cell lines, and in vivo, on five different human tumor xenotransplants. These include pancreatic cancer (PanC), TNBC and liver hepatocarcinoma (HC), and orthotopic metastatic colorectal cancer (CRC) model. Additionally, The-0504 activity was evaluated on a PDX model in comparison with the standard of care Nab-paclitaxel, in order to acquire information in view of potential clinical applications in PanC. Finally, The-0504 preliminary toxicity in mice and rats was investigated to assess its safety profile.

## Materials and methods

### The-0504 production

The-0504 was provided as lyophilized powder by Thena Biotech (Latina, Italy). The production and characterization of The-0504 is reported in a different paper [33].

### CD71 cell surface expression and The-05 cell uptake

CD71 cell surface expression was determined by fluorescence-activated cell sorting (FACS). Briefly, sub-confluent cells were detached using phosphate buffered saline (PBS)-ETDA 0.02%, washed with PBS-Bovine Serum Albumin (BSA) 0.2% and incubated (200,000 cells/tube) with anti-CD71 antibody or isotype control antibody conjugated to phycoerythrin (isotype-PE) (BD Biosciences, Milan, Italy) for 1 h at 4 °C. Finally, cells were washed with PBS-BSA 0.2% and cell-associated fluorescence was analyzed using a BD FACSCanto II apparatus (BD Biosciences).

The-05 cell uptake was also determined by FACS. To avoid the surface labeling of the protein nanocage, that could modify protein-protein interactions, we decided to load in the inner cavity of The-05 the antineoplastic drug Mitoxantrone, whose fluorescent spectra show two excitation peaks at 610 and 660 nm and an emission peak at 685 nm. Therefore, PaCa44 cancer cells were incubated with The-05-Mitoxantrone (The-05-Mit, 0.5 μM in Mitoxantrone) at 37 °C for different time intervals. After washing with PBS-BSA 0.2%, the fluorescence signal was measured using the APC channel of the BD FACSCanto II apparatus.

### Western blot analysis

Whole cell lysates were prepared using ice-cold lysis buffer supplemented with protease inhibitors. Protein content was determined by Bradford method. Twenty micrograms of total proteins were loaded on 10% sodium dodecyl sulfate polyacrylamide gel electrophoresis (SDS-PAGE) and transferred to a nitrocellulose membrane. The membrane was blocked with 5% not-fat dry milk in PBS with 0.01% Tween 20 for 1 h at room temperature and then incubated overnight with primary antibodies: anti-CD71 receptor (Abcam, Cambridge, UK) or anti-MMP-9 (Novus Biologicals, Centennial, USA) and anti-Glyceraldehyde 3-phosphate dehydrogenase (Cell Signaling Technology, Leiden, The Netherlands), all used at 1:1.000 dilution. The membrane was washed and incubated for 1 h at room temperature with the corresponding horseradish peroxidase-conjugated secondary antibody, diluted 1∶20,000 (BioRad, Milan, Italy). Bound antibodies were detected using the enhanced chemiluminescent (ECL) method (PerkinElmer Italia, Milan, Italy).

### The-0504 antiproliferative effects in vitro

Human cells from fibrosarcoma (HT1080), muscle rhabdomyosarcoma (A204), osteosarcoma (SJSA1), triple-negative breast (MDA-MB-231), colorectal (HT29), pancreatic (PaCa44, MiaPaCa2 and PanC-1), gastric (SNU-484) and liver (HepG2) cancer were grown in Roswell Park Memorial Institute (RPMI) Medium. All growth media were also added with 2 mM glutamine, 10% of fetal bovine serum (FBS) and antibiotics. Cancer cells (5 × 10^3^) were seeded in 90 μL of complete medium in 96-well culture microplates. The day after, cells were incubated in triplicate with 10 μL of serial dilutions of free Genz-644282 or The-0504. After 72 h-incubation at 37 °C with either the free drug or the nanotherapeutic agent, the medium was replaced with fresh medium w/o phenol red supplemented with XTT (2,3-Bis-(2-Methoxy-4-Nitro-5-Sulfophenyl)-2H-Tetrazolium-5-Carboxanilide) reagent (Sigma-Aldrich, St Louis, MO, USA), according to the manufacturer’s instructions. Finally, after a variable time ranging from 1 to 3 h of incubation at 37 °C, cell viability was measured at 450 nm by a microplate reader (VERSAmax, Molecular Devices, Sunnyvale, CA, USA). The percentage of cell viability was estimated by comparing cells treated with free Genz-644282 or The-0504 to mock treated cells. To compare free Genz-644282 and The-0504 killing efficacy, we evaluated the IC50, i.e., the drug or nanotherapeutic agent concentration yielding 50% cell viability.

### Animal models

Patient-derived xenografts (PDXs) were established by engrafting samples of primary pancreatic cancer obtained from patients by surgical resection, into the right flank of 4–6-week-old female CD1 nude mice (Charles River Laboratories; Calco, LC, Italy) as previously described by our group [36].

For the xenograft (subcutaneous) models, 4–6-week-old female CD1 nude mice (Charles River Laboratories; Calco, LC, Italy) were injected subcutaneously in the right flank with 3 × 10^6^ cells resuspended in 200 μl of PBS. When subcutaneous or PDX tumors reached a volume of about 80–100 mm^3^, mice were randomized in groups of six animals (four for Hep-G2) and injected i.v. with 200 μl of PBS, Genz-644282 or The-0504. The-0504 powder provided by Thena Biotech was reconstituted in water about one hour before administration. Genz-644282 was formulated in sodium lactate buffer as previously reported [34]. The treatment dose normalized to Genz-644282 concentration was 1.9 or 0.95 mg/Kg. Mice were treated twice a week for three weeks; tumor volume was measured with a caliper and mouse weight was monitored. In PDX model, Nab-paclitaxel, consisting of protein-bound paclitaxel particles for injectable suspension, was intravenously administered to the PDX model twice/weekly at the dose of 10 mg/K for a total of 3 weeks. Tumor volume of 1000–1500 mm^3^ was chosen as endpoint after which mice were sacrificed. Overall survival was also evaluated. For PaCa44 experiments, The-0504 treated mice that had survived beyond the observation period, were also subjected to magnetic resonance imaging (MRI) analysis to evaluate the presence of residual disease.

Before the analysis mice were anesthetized with isoflurane/oxygen. MRI images were acquired using a Biospec tomograph (Bruker, Karlsruhe, Germany) with a working field of 4.7 T and equipped with an actively shielded gradient system (Bruker) having a maximum gradient strength of 40 G/cm. For MRI, animals were placed in supine position in a 35 mm inner-diameter, birdcage coil. A sensor for breath monitoring was positioned at the level of the animal chest. Coronal T2-weighted images were acquired by using a fat-suppressed RARE sequence with the following parameters: TE = 56 ms, TR = 5000 ms, slice thickness = 0.1 cm, field of view (FoV) = 5.00 cm * 5.00 cm, NEX = 2.

For the orthotopic CRC mouse models, HT-29 cells stably expressing luciferase (1 × 10^6^ cells/mouse) were injected into the submucosal layer of the rectum of 4–6-week-old female CD1 nude mice (Charles River Laboratories; Calco, LC, Italy). In this mouse model the microinjection of tumor cells in the mucosa of the distal rectum not only recapitulates the primary tumor growth in the colon but also the tumor cell metastatization in pelvic lymph node, liver and lungs [37–39]. For surgical operations, the animals were anesthetized by 1.5% isoflurane inhalation in a mixture of oxygen and nitrogen. After anesthetization, the animals were positioned prone on a heated bed and with the anal orifice in front the operator. With a plastic microtube of 2.5 mm in diameter and using binocular lenses, to increase precision, 1 × 10^6^ HT-29 Luc + cells /100 uL were injected into the rectal submucosa using a 1 mL syringe with hand inclination angle of about 45 degrees with respect the horizontal plane of the surgery table. This surgical trans-anal cell injection made it possible to deposit HT-29 Luc + cancer cells directly into the rectal submucosa [40].

Bioluminescence imaging was used to follow tumor cell growth. Images were acquired prior to treatment and then weekly; mice were anesthetized with isoflurane/oxygen and injected i.p. with 150 mg/kg of D-luciferin (PerkinElmer Italia). After 10 min, tumor images were acquired with IVIS Spectrum Imaging System (PerkinElmer Italia). Five mice were imaged simultaneously, using the following parameters: exposure time = 1 min; view field = 18 cm, binning B = 8 and f/stop = 1. Living Image Software 4.4 (PerkinElmer Italia) was used for bioluminescence acquisition and quantification. In addition, to highlight the presence of small tumor foci spread in the upper abdomen and thoracic cavity, we have increased the signal of the apparatus during imaging and covered the high signals present in the large tumor burden of the perianal region.

### Immunohistochemistry in PDX tumors

CD71 receptor expression in PDX PANC#08 was evaluated using ex-vivo immunohistochemical analysis. After appropriate antigen retrieval procedure performance, slides were stained with anti-transferrin (CD71) receptor primary antibody (Abcam, Cambridge, UK) followed by the appropriate secondary antibodies. Immunoreactive antigens were detected using streptavidin peroxidase (Thermoscientific) and the DAB Chromogen System (Dako). After chromogen incubation, slides were counterstained in Hematoxylin (BioOptica).

### The-0504 therapeutic evaluation in vivo

The-0504 therapeutic activity evaluation was carried-out in three different animal facilities, using different cancer models. Experiments on human pancreatic PaCa44 and colorectal HT-29 cancer cells were conducted at the University of Verona (Italy). Experiments using Patient-Derived Xenograft (PDX) model and liver Hep-G2 cancer cells were performed at the University of Chieti (Italy). Experiments using triple-negative breast MDA-MB-231 cancer cells were conducted at the Regina Elena National Cancer Institute IRE, Rome (Italy).

Animal studies were performed according to a protocol approved by the Institutional Animal Care and Use Committee of the University of Verona, University of Chieti or IRE and authorized by the Italian Ministry of Health (Protocols no. 128/2014-B, 457/2018-PR and 89/2015-PR), and in accordance with the principles laid down in the European Community Council Directives (86/609/EEC).

### Response determination

Individual mice responses were determined as follows. Progressive disease (PD): tumor volume at the end of study, i.e., two weeks after the last treatment, is > 20% larger than the initial volume. Stable disease (SD): tumor volume is < 30% smaller than the initial volume during the study period and ≤ 20% larger than the initial volume two weeks after the last treatment. Partial response (PR): tumor volume is ≥30% smaller than the initial volume for at least one time-point and has a measurable tumor size (≥ 10 mm^3^). Complete response (CR): tumor disappearance (measurable tumor mass < 10 mm^3^) for at least one-time point. Maintained complete response: tumor volume < 10 mm^3^ at the end of the study period. The study period was set at 100 days from the beginning of therapy. For treated groups only, the percentage of tumor growth inhibition (% TGI) is defined as 100x(MTVcontrol-MTVtreated)/MTVcontrol, where MTV is the median tumor volume. The objective response rate (ORR) is defined as the percentage of treated animals that show a response (PR, CR or MCR) to therapy.

### Statistical analysis

A linear mixed-effects model was used to test the tumor volume change rate over time among different groups. Survival percentages were estimated using Kaplan-Meier methods, and survival curves were compared using the log-rank test.

### Multiple-cycle repeat-dose study in mice and rats

The objective of these studies was to preliminarily evaluate The-0504 in vivo toxicity. Female nude mice (*n* = 3) were subjected to repeated (q7dx4) intravenous (i.v.) injections, each followed by a two-week observation period. Two Genz-644282 equivalent dosages were used, i.e., 6 and 12 mg/kg. In addition, the empty carrier The-05 was also evaluated at 120 mg/Kg protein equivalent dosage (corresponding to about five times higher with respect to the 6 mg/Kg in Genz dosage). The Toxicity was evaluated based on body weight variation and clinical sign of distress appearance.

Rat experiments were performed by MTTlab (Trieste, Italy). Toxicity was determined by repeated (q7dx4) i.v. administration to male Wistar rat (*n* = 3), each followed by a two-week observation period. Four Genz-644282 equivalent dosages were used, i.e., 0.5; 1; 3; and 6 mg/kg. An additional group of rats, serving as negative controls, received the vehicle only. Before the start of the treatment, all rats were weighed; the weight range resulted to be 230–234 g. Animals were semi-randomly assigned to study groups in such a way that each group contained the same ratio of smaller and larger rats. Animals in each group were divided to 1 animal per cage. Cages were clearly labelled with an ID card indicating study number, group, gender and treatment schedule. Animals were weighed three times a week throughout the study and variations in body weight were recorded and used to adjust the dosage, if required. Animals were kept in a controlled environment, with a light/dark cycle of 12 h (h) each. All animals were subjected to the same environmental conditions. The study was authorized by the Italian Ministry of Health (Protocols no. 91/2019-PR) and carried out according to the guidelines enforced in Italy (DDL 116 of 21/2/1992 and subsequent addenda) and in compliance with the Guide for the Care and Use of Laboratory Animals, Department of Health and Human Services publication no. 86–23 (National Institutes of Health, Bethesda, MD, 1985).

At the end of the study all rats were culled by CO2 and gross autopsy was performed. Blood samples were collected from the heart, preferably the ventricle, by cardiac puncture. Thoracic and abdominal cavities were opened, and all major organs were macroscopically examined. Livers and kidneys from all animals were collected and weighted. Spleen, lungs, heart and femur were collected. Blood samples collected into EDTA containing tubes were subjected to hematological panel analysis, comprising: red blood cells counts (RBC); hematocrit; hemoglobin; mean corpuscular hemoglobin (MCH); mean corpuscular volume (MCV); reticulocyte counts; white blood cells counts (WBC); neutrophils; lymphocytes; and thrombocyte (platelet) counts. Blood samples collected into lithium heparin containing tubes were subjected to biochemical panel analysis, comprising: alanine aminotransferase or glutamate pyruvate transaminase (ALAT or GPT); albumin; globulin; albumin/globulin ratio; alkaline phosphatase; aspartate aminotransferase or glutamate oxaloacetate transaminase (ASAT or GOT); total bilirubin; calcium; chloride; creatinine; glucose; potassium; sodium; total protein; and urea.

Organs were collected from each animal and fixed with 10% formalin. Trimming procedures were carried out according to standard guidelines designed for toxicological pathology (https://reni.item.fraunhofer.de/reni/trimming/). Five-micron thick sections were stained with Haemotoxylin and Eosin (H&E) and evaluated under a light microscope. Histopathology evaluation was made in a blind fashion, i.e., in the absence of knowledge about the treatment group.

Femurs were collected into tubes containing physiological solution. Bone marrow analysis was conducted on the basis of scatter and immunophenotypic TBNK (T, B and natural killer subset) in flow cytometry: lymphocytes (TBNK-CD3 APC, CD45RA FITC, CD161a PE), monocytes-macrophages (only scatter) and relative counts by TrueCount counting beads.

Data analysis was performed by MS Excel and GraphPad Prism. Statistical analysis on kidneys and liver weight variation was performed using analysis of variance (ANOVA) and t-test. Results with confidence interval (CI) 95% (*p* < 0.05) were considered to be statistically significant.

## Results

### The-0504 design, production and characterization

Results on The-0504 design, production and characterization (Fig. 1) have been previously reported [33]. Overall, the The-0504 construct was found to be highly pure and monodispersed in solution, with mean diameter of about 18.0 nm and zeta-potential value of − 4.5 ± 0.9 mV [30, 33]. About 80 (81.0 ± 6.0) Genz-644282 molecules resulted to be stably entrapped in the internal cavity of The-0504. Free-drug release over time analysis indicates that the The-0504 protein-drug complex is highly stable (see Table S1).

### CD71 is expressed on the surface of GI and TNBC human cancer cells

**Fig. 2:**
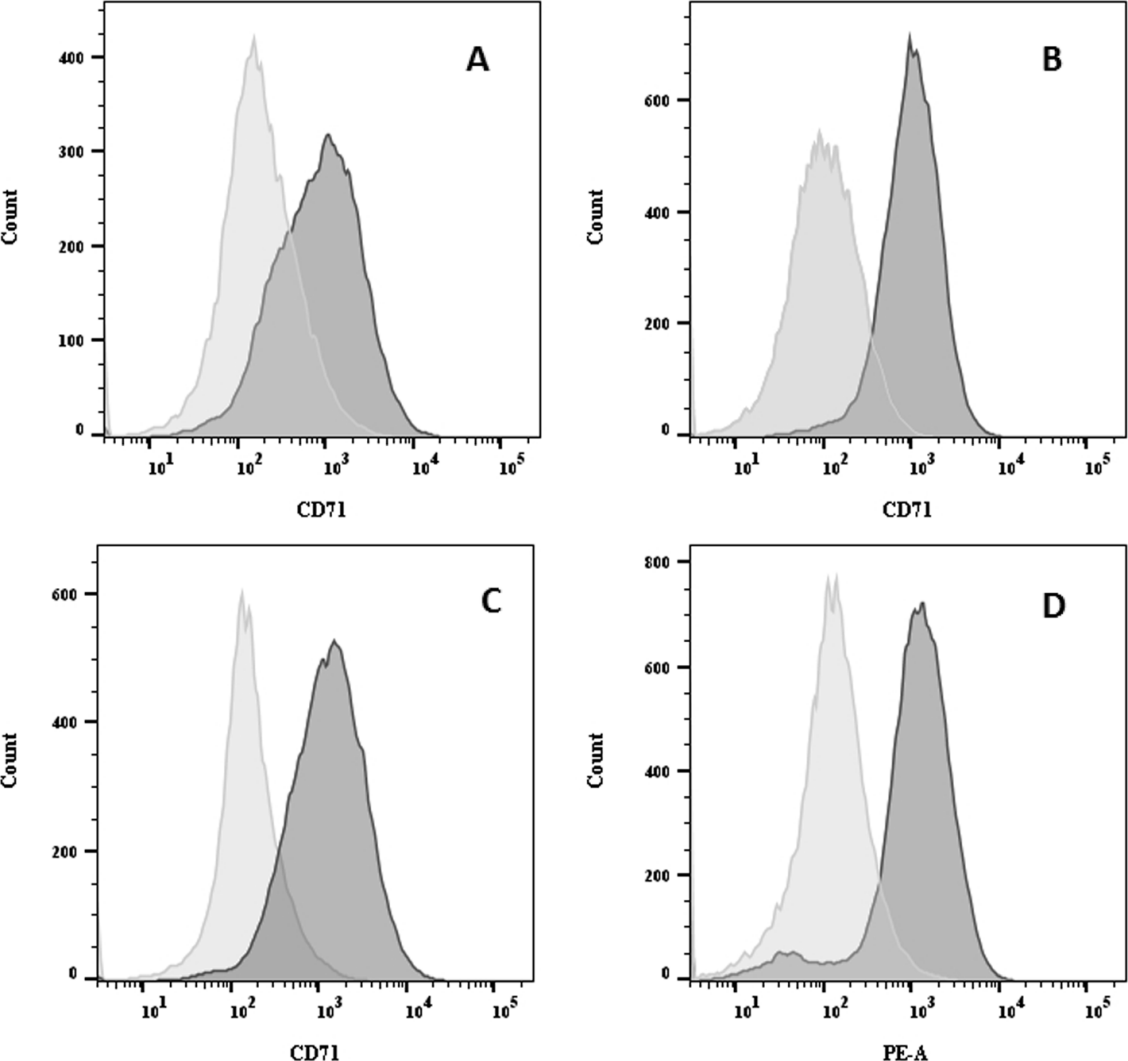
CD71 is highly expressed in cancer cells. Flow cytometry analysis of CD71 antigen expression on different cell lines: CD71 staining on HepG2 cells **a**; HT-29 cells **b**; MDA-MB-231 cells (**c**); and PaCa44 cells (**d**). Isotype-PE control staining is showed as transparent signal

CD71 plasma membrane expression was evaluated by FACS in a panel of human cancer cell lines (HepG2, HT-29, MBA-MD-231 and PaCa44), which were later used for in vivo experiments (see below). As depicted in Fig. 2, all of the investigated cancer cell lines showed high CD71 expression levels. In particular, HepG2, MDA-MB-231, PaCa44 and HT-29 cells showed a 5.7 ± 0.8, 9.1 ± 0.4, 8.2 ± 0.8 and 8.0 ± 1.7-fold increase in fluorescence signal with respect to control cells (isotype–PE antibody).

In addition to CD71 expression, we evaluated The-0504 ability to actually bind to the PaCa-44 cancer cell line. To this end, we took advantage of the ability of ferritin protein to encapsulate the fluorescent drug Mitoxantrone in its internal cavity, which has been previously reported by our group [30]. Therefore, the The-05 construct was loaded with Mitoxantrone (The-05-Mit), which acts as a fluorescence dye and whose emission in the APC channel can be measured by FACS. As shown in Fig. S1, median fluorescence intensity (MFI) values for The-05-Mit binding to PaCa44 cells were high and become more substantial after 360 min. These results agree with the previously reported high affinity of HFt-based constructs versus cancer cells.

### The-0504 is highly active against different tumor cell lines in vitro

To assess The-0504 ability to kill cancer cells in vitro, we performed XTT viability assays on a wide range of human cancer cell lines of different origin.

Results reported in Table 1 indicate that The-0504, whose internal cavity encapsulates Genz-644282, has IC50 values similar to those of free Genz-644282 in all tested cell lines, and even lower in some cases. This is remarkable, since naked drugs can freely diffuse into cells, whereas The-0504 can only deliver the encapsulated Genz-644282 following rate-limiting receptor-mediated uptake. The only exception occurred using the SJSA1osteosarcoma cell line. In this case, the killing ability of The-0504 was ten times lower with respect to the free drug. To investigate the reasons for this anomalous result, we evaluated both the MPP-9 and CD71 expression in this cell line by Western blot analysis (Fig. S2). We found that MMP-9, which is required for The-0504 unmasking, was expressed in the SJSA1 cell line (Fig. S2A), whereas CD71 expression was negligible (Fig. S2B). Therefore, the lower The-0504 cytotoxicity towards the SJSA1 cell line with respect to free Genz-644282 is more likely to be determined by reduced CD71-mediated The-0504 penetration than by lack of The-0504 unmasking.

**Table 1:** In vitro killing efficacy of free Genz-644282 and The-0504 against human cell lines of different origin. Values represent the mean ± SEM (*n* = 3)

***Cancer cell type***	***IC***_***50***_ ***(nM)*** ***Free Genz-644282***	***IC***_***50***_ ***(nM)*** ***The-0504***
HepG2	36.0 ± 7.1	23 ± 8.1
SNU-484	109.3 ± 36.9	75.7 ± 2.5
MDA-MB-231	19.03 ± 2.6	20.1 ± 1.7
A204	5.8 ± 0.41	4.44 ± 0.33
HT1080	5.4 ± 0.8	4.1 ± 0.5
SJSA-1	25.5 ± 3.8	251.7 ± 8.5
PaCa44	150.4 ± 30.6	100.4 ± 19.3
MIA PaCa-2	26.1 ± 2.6	14.5 ± 1.6
PANC-1	11.6 ± 1.3	14.2 ± 2.5
HT-29	160.3 ± 20.5	21.0 ± 2.1

### The-0504 is highly effective against xenograft models of pancreatic, breast and liver cancer

**Fig. 3:**
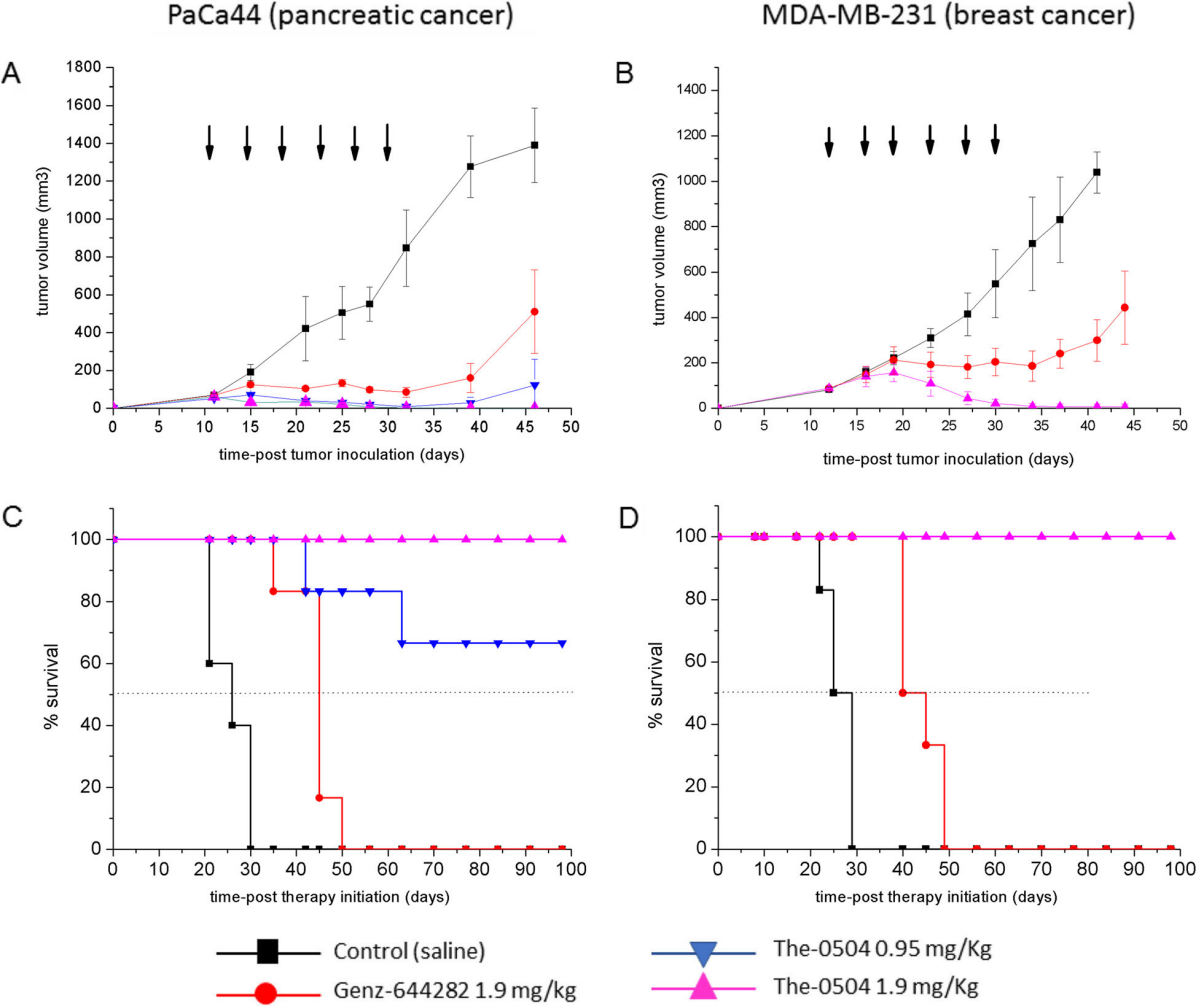
Anti-tumor activity of The-0504 in PaCa44 or MDA-MB-231 tumor-bearing mice. **a, b** Tumor-growth curves for each mouse group are indicated. Student’s t-test was used to determine statistical significance. **a** Control vs The-0504 0.95 mg/Kg: **p < 0.005; Control vs The-0504 1.9 mg/Kg: ***p < 0.0001; Control vs Genz-644282: *p < 0.05; Genz-644282 vs The-0504 1.9 mg/Kg: *p < 0.05. **b** Control vs The-0504: ***p < 0.0001; Genz-644282 vs The-0504 1.9 mg/Kg: **p < 0.005. **c, d** Survival curves of different animal groups. Mice were sacrificed when the tumor had reached a volume in the range 1000–1500 mm^3^. Statistical analysis was performed by log-rank test. **C** Control vs The-0504 0.95 mg/Kg, Control vs The-0504 1.9 mg/Kg and Control vs Genz-644282: **p < 0.005; Genz-644282 vs The-0504 0.95 mg/Kg: *p < 0.05; Genz-644282 vs The-0504 1.9 mg/Kg: **p < 0.005. **d** Control vs The-0504: **p < 0.005; Genz-644282 vs The-0504: **p < 0.005. Arrows indicate the six days during which treatments were administered

The-0504 anticancer activity was initially evaluated in CD71 expressing subcutaneous xenografts of pancreatic (PaCa44) and triple-negative breast (MDA-MB-231) cancer cells (Fig. 2). A preliminary evaluation of hepatocarcinoma (HepG2) model was also carried-out and reported as supplementary information (Fig. S3). Tumor-bearing animals were randomized when the tumor was about 80–100 mm^3^ and treated with free Genz-644282 and The-0504, both at 1.9 mg/kg in Genz-644282, twice a week for three consecutive weeks by intravenous injections. For PaCa44 experiment, an exploratory half-dose of 0.95 mg/Kg in Genz-644282 was also evaluated to explore different regimes for potential future applications. As shown in Fig. 3, both PaCa44 and MDA-MB-231 tumor growth was significantly inhibited in mice treated with 1.9 mg/kg free Genz-644282; conversely, in control groups, tumors grew rapidly and reached a size > 1000 mm^3^ at days 35–40 after tumor cell injection. However, tumor growth stalled only during the three-week period treatment with Genz-644282, and resumed as soon as treatment was discontinued. Tumor Growth Inhibition (TGI) and Overall Response Rate (ORR) values for free Genz-644282 were 64.2 and 16.6%, respectively, in PaCa44 experiment and 70.1 and 0%, respectively, in MDA-MB-231 experiment (Table 2). In contrast, The-0504 led to long-term regression of all established tumors, with 100% values of both TGI and ORR (Table 2), in both PaCa44 and MDA-MB-231 experiments. Of note, in PaCa44 experiment, the The-0504 half-dose (i.e., 0.95 mg/Kg) was also highly effective, with TGI and ORR values of 92.8 and 100%, respectively (Table 2), and 66.6% of disease-free animals. As shown in Fig. 3c-d, the survival of The-0504-treated groups exceeded the observation period of 100 days in both PaCa44 and MDA-MB-231 experiments, whereas the median survival of Genz-644282 treated-groups was 42.5 and 40 days in PaCa44 and MDA-MB-231 mouse models, respectively.

**Table 2:** Summary of Genz-644282 and The-0504 efficacy in tumor-bearing mice models of human cell lines of different origin

Tumor model	TGI (%)		RESPONSE at 1.9 mg/Kg	
	ORR (%)			
	*Genz-644282*	*The-0504*	*Genz-644282*^a^	*The-0504*
Pancreatic (PaCa44)	64.2	100	33.3% SD	100% MCR
	16.7	100	66.7% PD	
Colorectal (HT29)	*Not available*	*Not available*	33.3% SD	66.7% CR
	16.6	100	66.7% PD	33.3% PR
Breast (MDA-MB 231)	70.1	100	16.7% SD	83.3% MCR
	0	100	83.3% PD	16.7% CR
Liver (HepG2)	55.5	100	25.0% PR	100% MCR
	25.0	100	75.0% PD	
Pancreatic (PDX)^a^	0	93.6	100% PD	33.3% MCR
	0	83.3		16.7% CR,
				33.3% PR,
				16.7% SD

^a^ Nab-paclitaxel at 10 mg/Kg instead of Genz-644282 was used in PDX model

*TGI* Tumor Growth Inhibition, *ORR* Objective Response Rate, *MCR* Maintained Complete Response, *CR* Complete Response, *PR* Partial Response, *SD* Stable Disease, *PD* Progressive Disease

The absence of any residual sign of disease in PaCa44 experiment mice treated with The-0504 at 1.9 mg/Kg was assessed by magnetic resonance imaging (MRI). Neither subcutaneous thickening nor tumor masses were observed, confirming that 100% of animals were completely cured of the tumor (Fig. S4).

Similar results were obtained in a preliminary hepatocarcinoma (HepG2) model experiment using a lower number of animals/group (*n* = 4) (Fig. S3). In this model, TGI and ORR values were 55.5 and 25.0%, respectively, following treatment with free Genz-644282, and 100% both following treatment with The-0504 (Table 2), indicating that the impressive in vivo cytotoxic activity of this new therapeutic agent has a broader scope than pancreas and breast cancers.

### The-0504 is highly effective in an orthotopic metastatic colorectal cancer (CRC) model

**Fig. 4:**
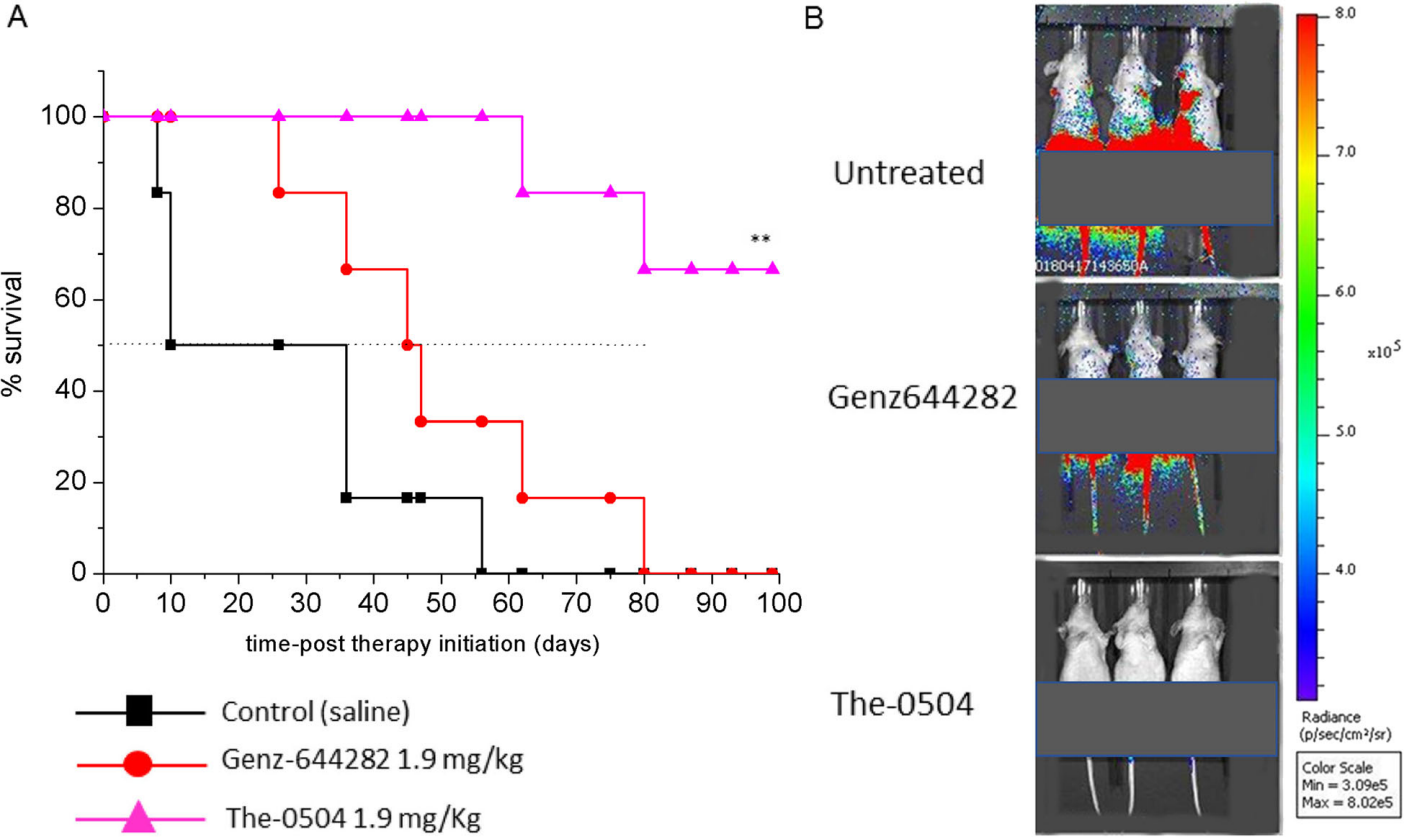
Anti-tumor activity of The-0504 in mice bearing orthotopic HT-29 tumors. **a** Survival curves of different animal groups. Animals were sacrificed when they showed signs of pain or cachexia. Statistical analysis was performed by log-rank test. Control vs The-0504: **p < 0.005; Genz-644282 vs The-0504: *p < 0.05. **b** Representative bioluminescence images of HT-29 lung metastases. Images of three mice/group 10 days after the last treatment (i.e., 30 days after therapy start) are shown. Primary tumors are covered by a black paper to avoid possible camera saturation

In order to evaluate The-0504 activity in a highly metastatic orthotopic CRC mouse model, HT-29 cells stably expressing luciferase were injected into the submucosal layer of the rectum (see the Methods section for model generation details). Activity was compared with that of equivalent amounts of free Genz-644282. Bioluminescence imaging was used to monitor both HT-29 cell expansion at the primary site and the appearance of metastatic pulmonary disease. Free Genz-644282 increased survival by 35 days compared to mock treated mice, with a median survival of 45 days (Fig. 4a). The-0504 treatment was significantly more effective, since 66.6% of mice showed complete response and were alive at the end of the study period of 100 days. ORR values for free Genz-644282- and The-0504-treated mice were 16.6 and 100%, respectively (Table 2). It is worth pointing out that metastatic loci were completely absent in the The-0504 group 10 days after the last treatment, i.e., 30 days since therapy start, in contrast to both controls and free Genz-644282 treated animal groups (Fig. 4b).

### The-0504 is more effective than the standard of care nab-paclitaxel against patient-derived xenograft models of pancreatic cancer

The results from PaCa44, MDA-MB-231, HepG2 and HT29 in vivo experiments prompted us to compare The-0504 activity with a standard of care drug for pancreatic cancer, in a clinically relevant model. For this reason, an experiment was undertaken in nude mice with a Patient-Derived Xenograft (PDX) model of pancreatic cancer, generated as described in the methods section [36]. As shown in Fig. S5, an immunohistochemical–based assay revealed that CD71 is highly expressed in this model.

**Fig. 5:**
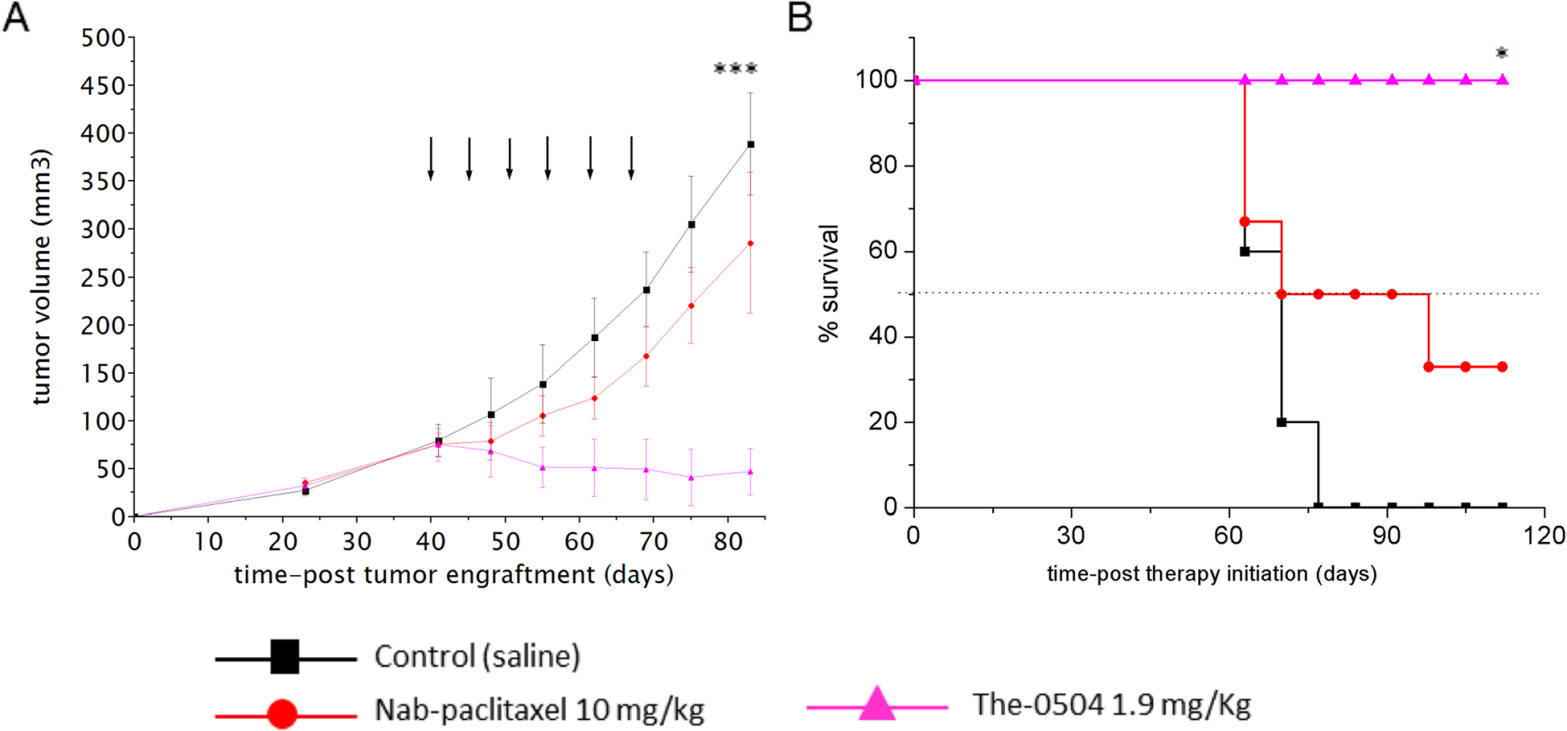
Anti-tumor activity of The-0504 in a pancreatic cancer PDX model. **a** Tumor-growth curves for each mouse group are indicated. Student’s t-test was used to determine statistical significance. Control vs The-0504 and Nab-paclitaxel vs The-0504: ***p < 0.0001. **b** Survival curves of different animal groups. Animals were sacrificed when the tumor had reached a volume of about 1000 mm^3^. Statistical analysis was performed by log-rank test. Control vs The-0504: *p ≤ 0.03; Nab-paclitaxel vs The-0504: *p ≤ 0.03. Arrows indicate the six days during which treatment was administered

The therapeutic activity of 1.9 mg/Kg The-0504 was compared with that of 10 mg/Kg Nab-paclitaxel, a nanoformulation of the paclitaxel drug. This Nab-paclitaxel dose regimen was previously reported to produce a 72% of tumor growth inhibition using AsPC-1 pancreatic tumor bearing mice [41]. In our PDX model, the tumor growth was not significantly inhibited by Nab-paclitaxel but only showed a moderate delay, whereas The-0504 was highly effective (Fig. 5a). TGI and ORR values were 20.0 and 0%, respectively, for Nab-paclitaxel and 93.6 and 83.3%, respectively, for The-0504 (Table 2). In accordance with these values, all The-0504 administered mice survived during the observation period of 115 days from therapy start (Fig. 5b). Only one mouse died at day 55 after tumor implantation for a technical problem during drug injection (embolia), and it was not included in the survival evaluation shown in Fig. 5b.

### The-0504 is well tolerated in both mice and rats

The-0504 toxicity in mice and rats was assessed by repeated (q7dx4) intravenous (i.v.) administration to female CD1 nude mice (*n* = 3) or male Wistar rats (*n* = 3), followed by a two-week observation period.

Two different doses were used as Genz-644282 equivalents: 6 and 12 mg/kg in mice, and 3 and 6 mg/kg in rats, using an interspecies dose conversion table reported in [42]. No treatment-related toxicities were observed in mice treated with 6 mg/Kg The-0504, as demonstrated by the absence of significant body weight loss (Fig. S6). In contrast, two out of three mice treated with 12 mg/Kg The-0504 showed significant body weight loss and died after the second injection. Therefore, 6 mg/Kg was identified as a safe for The-0504 drug in mouse. Similarly, a dose of 4 mg/Kg was indicated as the maximum tolerated dose for free Genz-644282 by other groups [43]. As expected and in accordance with previous results reported for the empty native ferritin [44], no toxicities were observed also in mice treated with the empty The-05 carrier at 120 mg/Kg, equivalent to 30 mg/Kg Genz-644282 dosage (not shown).

In experiments with rats, blood analyses and organ histopathology were performed in addition to general examination and body weight evaluation. Results are reported in the supplementary information section. Similar to what observed in experiments with mice, no obvious compound-related adverse effects were observed with the lowest (3 mg/Kg) The-0504 dose. The body weight of all animals steadily increased until the end of the study, with final body weight gain of + 48.4% and + 43.2% in control group (G1) and in the group treated with 3 mg/Kg The-0504 (G2), respectively (Fig. S7). Blood analyses of G1 and G2 groups provided very similar results (Fig. S8). The final macroscopic examination of thoracic and abdominal cavity and major organs did not reveal any abnormalities and all organs were of usual size and weight. Average weight of cleaned right kidney (AVG ± SEM) was 1.22 ± 0.04 and 1.13 ± 0.03 g for groups G1 and G2, respectively. Average weight of cleaned left kidney (AVG ± SEM) was 1.19 ± 0.058 and 1.17 ± 0.033 g for groups G1 and G2, respectively. The kidney weight was not significantly different (CI 95%) between the groups G1, G2 or left and right-side organs. Average weight of cleaned liver (AVG ± SEM) was 13.37 ± 0.745 and 13.43 ± 0.296 g for groups G1 and G2, respectively. The liver weight was not significantly different between G1 and G2 groups (CI 95%). Severe lesions of toxicological significance were not observed. Only a mild extramedullary hematopoiesis (EMH) of the erythroid type was observed in the spleen of the G2 group (Fig. S9). Other histological findings observed in the examined organs are common incidental findings in laboratory rats, and should not be considered to be treatment related. Bone marrow analysis was conducted as described in the Methods section. No significant difference in G1 and G2 groups bone marrow population was observed (Fig. S10). Therefore, 3 mg/Kg was identified as the safe dose for The-0504 in rat. Again, similar to what observed in experiments with mice, rats treated with the highest (i.e., 6 mg/Kg) The-0504 dose showed significant loss in body weight and were sacrificed after two weeks after treatments start (Fig. S7). Consequently, this dose was considered to be not tolerated for The-0504 drug in rats.

## Discussion

In this work, we report the preclinical evaluation of a novel protein nanocarrier:drug complex, named The-0504, which is endowed with high tumor killing efficacy and low toxicity in preclinical studies.

As demonstrated by experiments carried out in three different animal facilities, The-0504 is able to cure 100% of mice bearing different types of aggressive solid tumors, such as pancreatic, triple-negative breast and liver cancer, and is even able to produce 100% objective response rates (66.7% complete responses and 33.3% partial responses) in treated mice with highly metastatic CRC and 83.3% objective response rates (50% complete responses and 33.3% partial responses) in treated mice bearing PDX pancreatic cancer model (Table 2). Additionally, toxicology studies in mice and rats revealed that The-0504 had satisfactory safety profiles of The-0504, particularly upon chronic treatment (Fig. S6, S7, S8, S9 and S10). The-0504 has also been observed to have high tolerability in preliminary studies involving non-human primates (data not shown).

The high The-0504 anticancer activity reported in this work can be ascribed to its remarkable selectivity for cancer cells that express high levels of CD71 with respect to normal cells, as demonstrated by the absence of treatment-related toxicity in mice and rats at clinically-relevant doses. The-0504 high selectivity is contributed by two main factors. First, the targeted CD71 receptor is expressed at low levels, although virtually ubiquitously, in normal cells, and at high levels by the large majority of existing cancer types [24]. Accordingly, all cancers investigated in this work (i.e., pancreatic, triple negative breast, liver and colorectal cancer) that express high CD71 levels are more sensitive to The-0504 than free Genz-464,282 (Fig. 2 and S4); only the SJSA1 osteosarcoma cell line, which has low CD71 expression levels, is killed less effectively by The-0504 than by the free drug (Table 1 and Fig. S2). Second, The-0504 can only efficiently bind the target CD71 receptor following HFt unmasking by tumor-specific proteases. Indeed, within the The-0504 construct, the HFt external surface is joined with a specific masking polypeptide, which effectively dampens CD71 binding to normal tissues. The masking polypeptide and HFt are joined via a peptide sequence that can only be removed by cancer-specific proteases MMP-2/9, selectively expressed in the tumor microenvironment [29, 30]. As previously reported by our group, this double CD71/MMP targeting strategy, achieved by a masking/unmasking mechanism, was responsible for reduced unwanted effects, as well as prolonged blood half-life [29, 30].

The combination of the targeting strategy with high-density cytotoxic compound incorporation in a single product, allowed The-0504 to overcome some of the limitations of previously reported CD71-targeting agents. For example, anti-CD71 antibodies have been proved to be too toxic, due to the widespread distribution of the receptor [45]; on the other hand, none of the previously reported HFt variants entrapping cytotoxic compounds has been able to induce complete tumor regressions [8].

Of note, The-0504 outperformed Nab-paclitaxel (Fig. 5 and Table 2), the only cancer nano-therapeutic approved so far, and now standard-of-care in the treatment of pancreatic cancer.

Taken together, The-0504 high efficacy and low toxicity in several preclinical cancer models indicate that this nanotherapeutic agent may be an effective weapon to add to the arsenal of therapeutic tools currently used against human cancers. Regulatory studies are ongoing in rats and non-human primates to conclusively demonstrate The-0504 safety.

## Conclusions

In conclusion, the evidence reported in this work strongly suggests that the The-0504 ferritin-based nanocarrier is actually capable of low toxicity and high efficacy CD71-targeting, and it is a versatile platform to entrap and selectively redirect wide-spectrum anti-tumor agents that would be too toxic to be used in their naked form in the clinic.

## Supplementary Information

**Additional file 1: Table S1.** Free-drug content in lyophilized The-0504 after storage at 2–8 °C for eight months. **Fig. S1.** The-05 binding to pancreatic PaCa44 cells. Flow cytometric analysis of The-05. Mitoxantrone-containing The-05 (The-05-Mit) preparation was incubated for different times at 37 °C with human PaCa44 cells. Binding was revealed by direct fluorescence reading in a BD FACSCanto II flow cytometer. The-05-Mit provided at the predetermined optimal concentration of 0.5 μM (in Mitoxantrone). MFI refers to the median fluorescence intensity of the sample subtracted from the cell autofluorescence value. **Fig. S2.** MMP-9 and CD71 expression evaluation in cancer cells. Western blot using anti-MMP-9 antibody on SJSA-1 cells (A) or anti-CD71 antibody on SJSA-1, Panc-1 and MIA-PaCa-2 cells (B). GAPDH was used as a loading control. **Fig. S3.** Anti-tumor activity of The-0504 in mice bearing subcutaneous HepG2 liver tumors. Tumor-growth curves for each mouse groups are indicated. Animals were observed up to 60 days. At that time, 100% of The-0504-treated mice were still alive and with any residual sign of disease. Statistical significance according to the Student’s t-test: control vs The-0504 * *p* < 0.05, Genz-644282 vs The-0504 * *p* < 0.05. Arrows indicate the six The-0504 administrations. **Fig. S4.** Representatives Magnetic Resonance Images of mice treated with The-0504 at sacrifice. Mice with established subcutaneous PaCa44 tumors treated with The-0504 (1.9 mg/Kg) were imaged at the end of 100 days study period. MRI images acquired just before the sacrifice, evidencing the substantial absence of tumor mass on the skin of mice. **Fig. S5.** Transferrin receptor (CD71) expression on PDAC tumor. Immunohistochemical analysis of PDAC parental tumor from patient PANC#08 showing CD71 high expression. **Fig. S6.** Body weight in healthy mice after The-0504 administration. The-0504 was injected intravenously once a week for four weeks. Mouse body weight was measured twice a week. Control (*n* = 3); The-0504 6 mg/Kg (n = 3); The-0504 12 mg/Kg (n = 3). After the second injection, only one mouse of The-0504 12 mg/Kg group survived and was monitored until the end of the experiment. **Fig. S7.** Body weight in Wistar rats after The-0504 administration. The-0504 was injected intravenously once a week for four weeks. Rat body weight was measured three times a week. Control (n = 3); The-0504 3 mg/Kg (n = 3); The-0504 6 mg/Kg (n = 3). All rats of The-0504 6 mg/Kg group were sacrificed after two weeks from the treatments start due to significant body weight loss. **Fig. S8.** Blood sample analysis in Wistar rats after The-0504 administration at 3 mg/Kg. Hematological (top) and biochemical (bottom) analysis after The-0504 treatment in Wistar rats. **Fig. S9.** Representatives images of immunohistochemistry (IHC). IHC of control (left) and 3 mg/Kg The-0504 (right) treated rats. Organs are indicated. **Fig. S10.** Bone marrow cell counts in Wistar rats after The-0504 administration at 3 mg/Kg. Absolute (top) and relative percentages (bottom) regarding WBC total cells (blue), granulocytes (red), monocytes (gray) and lymphocytes (yellow). Results show a slight increase in WBC population in treated group (rats 4–6) in comparison to control group (rats 1–3) and no differences in the other BM populations.

## Data Availability

All data supporting the findings of this study are available within this paper and from the corresponding authors.

## Abbreviations

CD: Cluster of differentiation
TfR1: Transferrin receptor 1
HFt: Human ferritin heavy chain
TNBC: Triple-negative breast cancer
GI: Gastrointestinal cancers
HER2: Human epidermal growth factor receptor 2
MMP: Matrix-metalloproteases
IC50: Half maximal inhibitory concentration
PanC: Pancreatic cancer
HC: Liver hepatocarcinoma
CRC: Colorectal cancer
PBS: Phosphate buffered saline
BSA: Bovine Serum albumin
PE: Phycoerythrin
SDS-PAGE: Sodium dodecyl sulfate polyacrylamide gel electrophoresis
ECL: Enhanced chemiluminescent
RPMI: Roswell Park Memorial Institute
FBS: Fetal bovine serum
XTT: 2,3-Bis-(2-Methoxy-4-Nitro-5-Sulfophenyl)-2H-Tetrazolium-5-Carboxanilide
PDX: Patient-derived xenografts
MRI: Magnetic resonance imaging
i.p.: Intraperitoneal
i.v.: Intravenous
TGI: Tumor Growth Inhibition
ORR: Objective Response Rate
MCR: Maintained Complete Response
CR: Complete Response
PR: Partial Response
SD: Stable Disease
PD: Progressive Disease
TGI: Tumor growth inhibition
MTV: Median tumor volume
ORR: Objective response rate
RBC: Red blood cells
MCH: Mean corpuscular hemoglobin
MCV: Mean corpuscular volume
WBC: White blood cell counts
ALT: Alanine aminotransferase
GPT: Glutamate pyruvate transaminase
ASAT: Aspartate aminotransferase
GOT: Glutamate Oxaloacetate Transaminase
H&E: Haemotoxylin and Eosin
APC: Antigen presenting cells
RA: Retinoic acid
FITC: Fluorescein isothiocyanate
CI: Confidence interval
FACS: Fluorescence-activated cell sorting
FL: Fluorescent label
MFI: Median fluorescence intensity
SEM.: Standard error of mean
GC: Control group
AVG: Average
CI: Confidence interval
EMH: Extramedullary hematopoiesis
