# Supplementary material for: High activity and low toxicity of a novel CD71-targeting nanotherapeutic named The-0504 on preclinical models of several human aggressive tumors
Source: J Exp Clin Cancer Res. 2021 Feb 10;40:63. doi: 10.1186/s13046-021-01851-8 (PMC7877078; doi:10.1186/s13046-021-01851-8)
Supplement: Supplementary file 1 — Additional file 1: Table S1. Free-drug content in lyophilized The-0504 after storage at 2–8 °C for eight months. Fig. S1. The-05 binding to pancreatic PaCa44 cells. Flow cytometric analysis of The-05. Mitoxantrone-containing The-05 (The-05-Mit) preparation was incubated for different times at 37 °C with human PaCa44 cells. Binding was revealed by direct fluorescence reading in a BD FACSCanto II flow cytometer. The-05-Mit provided at the predetermined optimal concentration of 0.5 μM (in Mitoxantrone). MFI refers to the median fluorescence intensity of the sample subtracted from the cell autofluorescence value. Fig. S2. MMP-9 and CD71 expression evaluation in cancer cells. Western blot using anti-MMP-9 antibody on SJSA-1 cells (A) or anti-CD71 antibody on SJSA-1, Panc-1 and MIA-PaCa-2 cells (B). GAPDH was used as a loading control. Fig. S3. Anti-tumor activity of The-0504 in mice bearing subcutaneous HepG2 liver tumors. Tumor-growth curves for each mouse groups are indicated. Animals were observed up to 60 days. At that time, 100% of The-0504-treated mice were still alive and with any residual sign of disease. Statistical significance according to the Student’s t-test: control vs The-0504 * p < 0.05, Genz-644282 vs The-0504 * p < 0.05. Arrows indicate the six The-0504 administrations. Fig. S4. Representatives Magnetic Resonance Images of mice treated with The-0504 at sacrifice. Mice with established subcutaneous PaCa44 tumors treated with The-0504 (1.9 mg/Kg) were imaged at the end of 100 days study period. MRI images acquired just before the sacrifice, evidencing the substantial absence of tumor mass on the skin of mice. Fig. S5. Transferrin receptor (CD71) expression on PDAC tumor. Immunohistochemical analysis of PDAC parental tumor from patient PANC#08 showing CD71 high expression. Fig. S6. Body weight in healthy mice after The-0504 administration. The-0504 was injected intravenously once a week for four weeks. Mouse body weight was measured twice a week. Control ( [file 13046_2021_1851_MOESM1_ESM.docx]

Supplementary Material:

**High activity and low toxicity of a novel CD71-targeting nanotherapeutic named The-0504 on preclinical models of several human aggressive tumors**

**Authors:** Elisabetta Falvo^*^, Verena Damiani, Giamaica Conti, Federico Boschi, Katia Messana, Patrizio Giacomini, Michele Milella, Vincenzo De Laurenzi, Veronica Morea, Gianluca Sala, Giulio Fracasso^*^, Pierpaolo Ceci

*To whom correspondence should be addressed: elisabetta.falvo@cnr.it and giulio.fracasso@univr.it

**SUPPLEMENTARY TABLE S1**

**Table S1. Free-drug content in lyophilized The-0504 after storage at 2–8 °C for eight months.**

| **Time (months)** | **Free-Genz644282 (%)** |
| --- | --- |
| 0 | 5.2 |
| 2 | 5.8 |
| 4 | 6.7 |
| 6 | 6.3 |
| 8 | 6.1 |

**SUPPLEMENTARY FIGURES S1-S10**


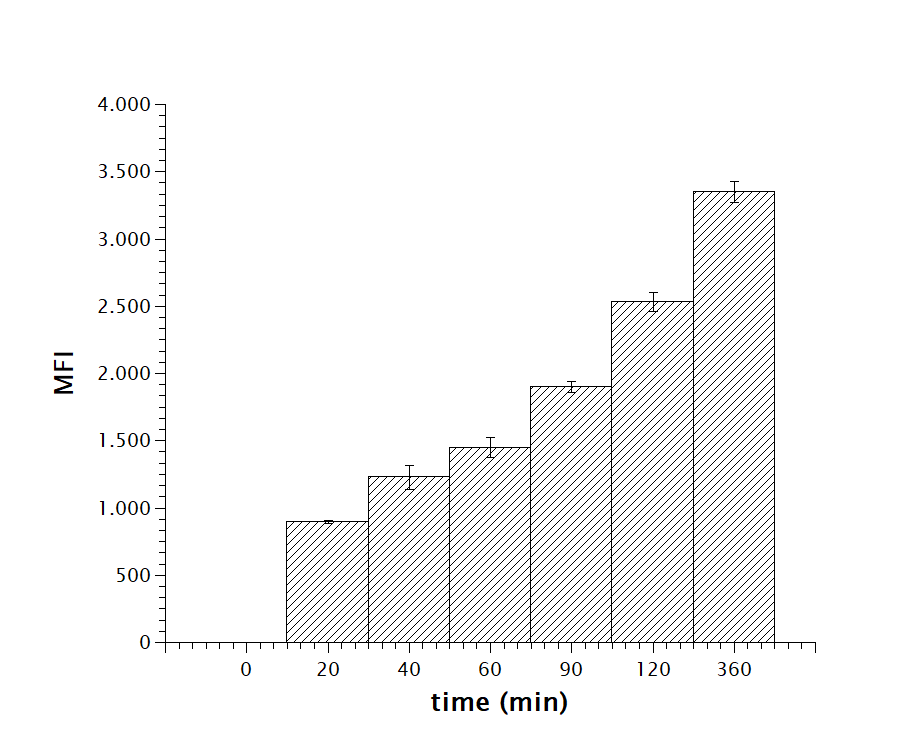


**Fig. S1. The-05 binding to pancreatic PaCa44 cells.** Flow cytometric analysis of The-05. Mitoxantrone-containing The-05 (The-05-Mit) preparation was incubated for different times at 37 °C with human PaCa44 cells. Binding was revealed by direct fluorescence reading in a BD FACSCanto II flow cytometer. The-05-Mit provided at the predetermined optimal concentration of 0.5 µM (in Mitoxantrone). MFI refers to the median fluorescence intensity of the sample subtracted from the cell autofluorescence value.


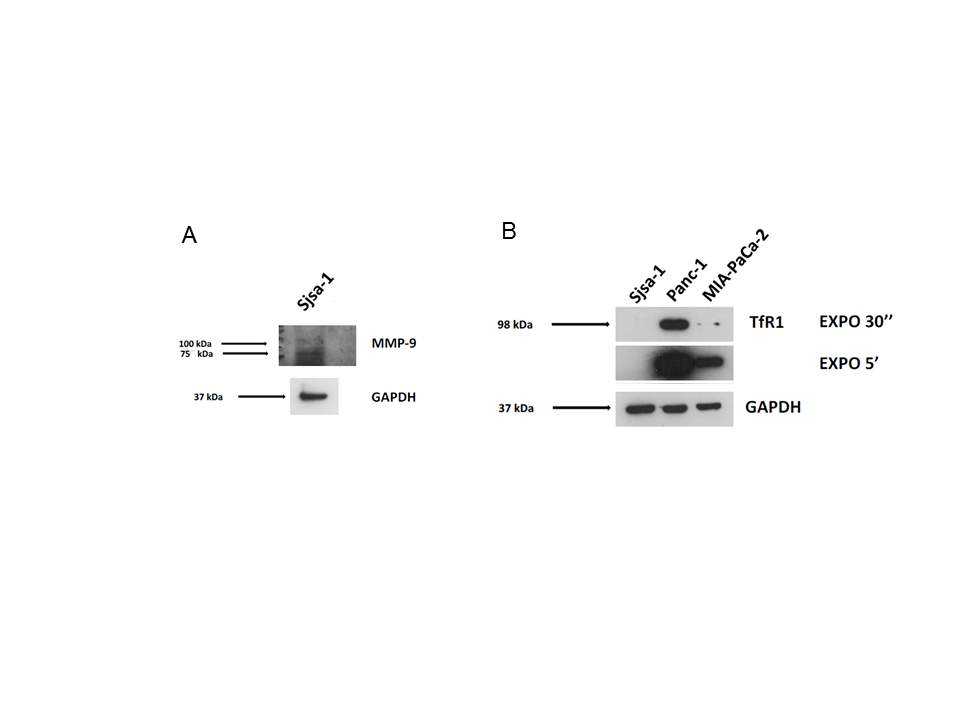


**Fig. S2. MMP-9 and CD71 expression evaluation in cancer cells.** Western blot using anti-MMP-9 antibody on SJSA-1 cells (A) or anti-CD71 antibody on SJSA-1, Panc-1 and MIA-PaCa-2 cells (B). GAPDH was used as a loading control


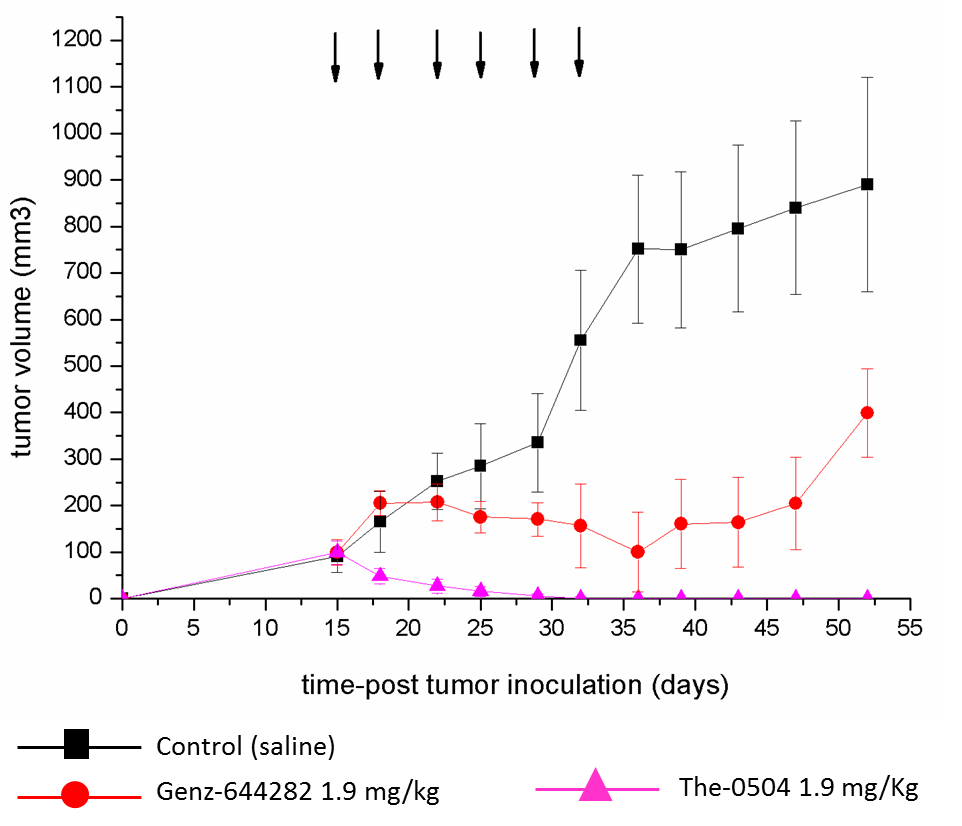


**Fig. S3. Anti-tumor activity of The-0504 in mice bearing subcutaneous HepG2 liver tumors**. Tumor-growth curves for each mouse groups are indicated. Animals were observed up to 60 days. At that time, 100% of The-0504-treated mice were still alive and with any residual sign of disease. Statistical significance according to the Student's t-test: control vs The-0504 * p < 0.05, Genz-644282 vs The-0504 * p < 0.05. Arrows indicate the six The-0504 administrations.

**
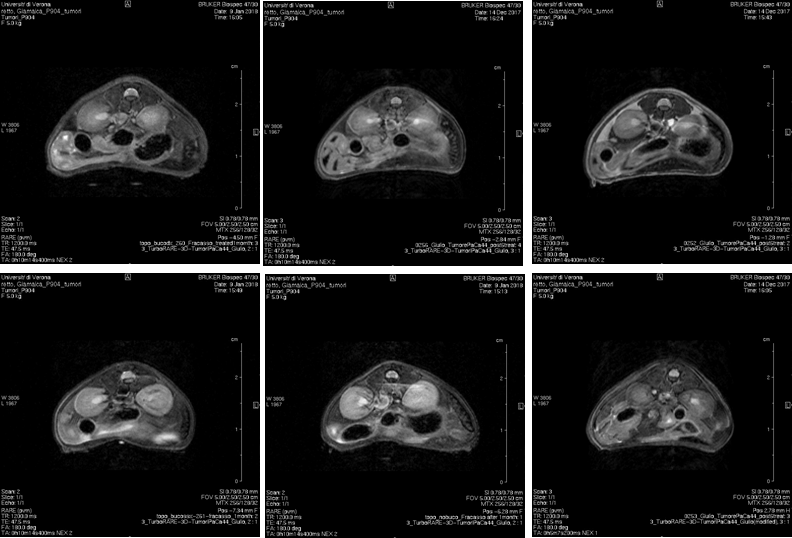
**

**Fig. S4. Representatives Magnetic Resonance Images of mice treated with The-0504 at sacrifice.** Mice with established subcutaneous PaCa44 tumors treated with The-0504 (1.9 mg/Kg) were imaged at the end of 100 days study period. MRI images acquired just before the sacrifice, evidencing the substantial absence of tumor mass on the skin of mice.

**
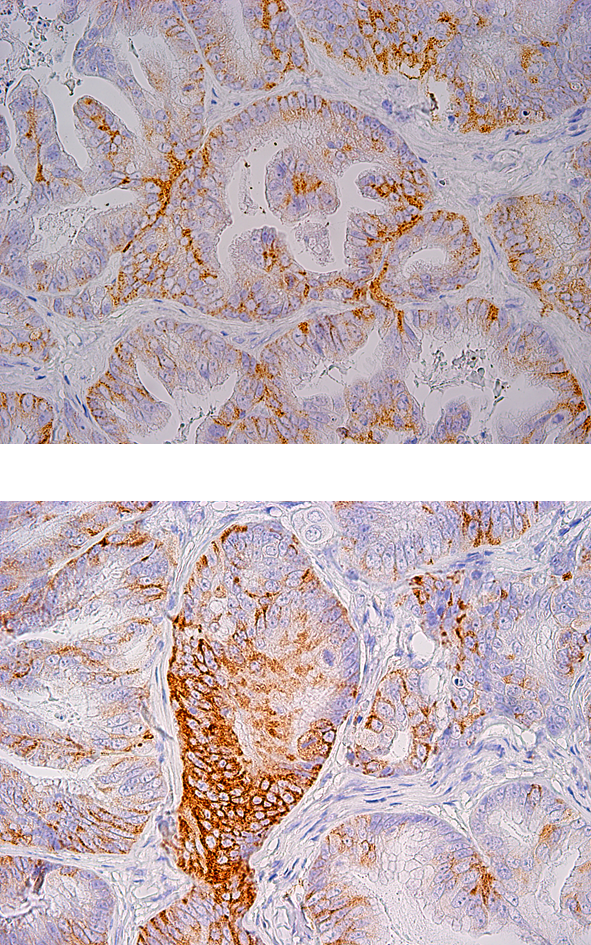
**

**Fig. S5. Transferrin receptor (CD71) expression on PDAC tumor.** Immunohistochemical analysis of PDAC parental tumor from patient PANC#08 showing CD71 high expression.


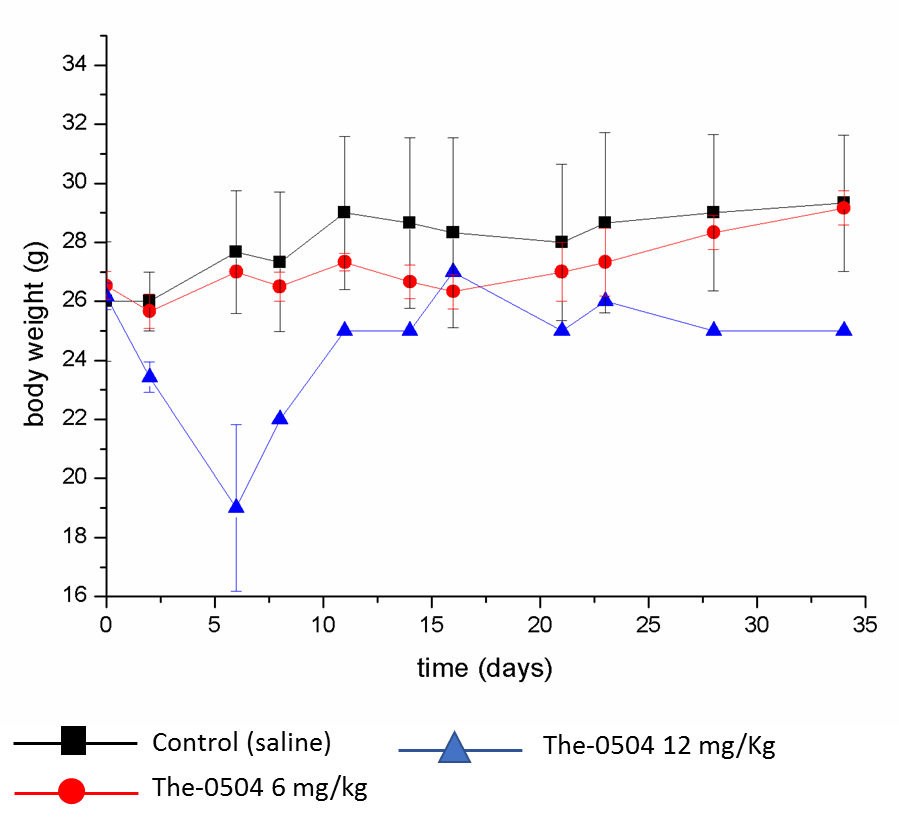


**Fig. S6. Body weight in healthy mice after The-0504 administration**. The-0504 was injected intravenously once a week for four weeks. Mouse body weight was measured twice a week. Control (n=3); The-0504 6 mg/Kg (n=3); The-0504 12 mg/Kg (n=3). After the second injection, only one mouse of The-0504 12 mg/Kg group survived and was monitored until the end of the experiment.


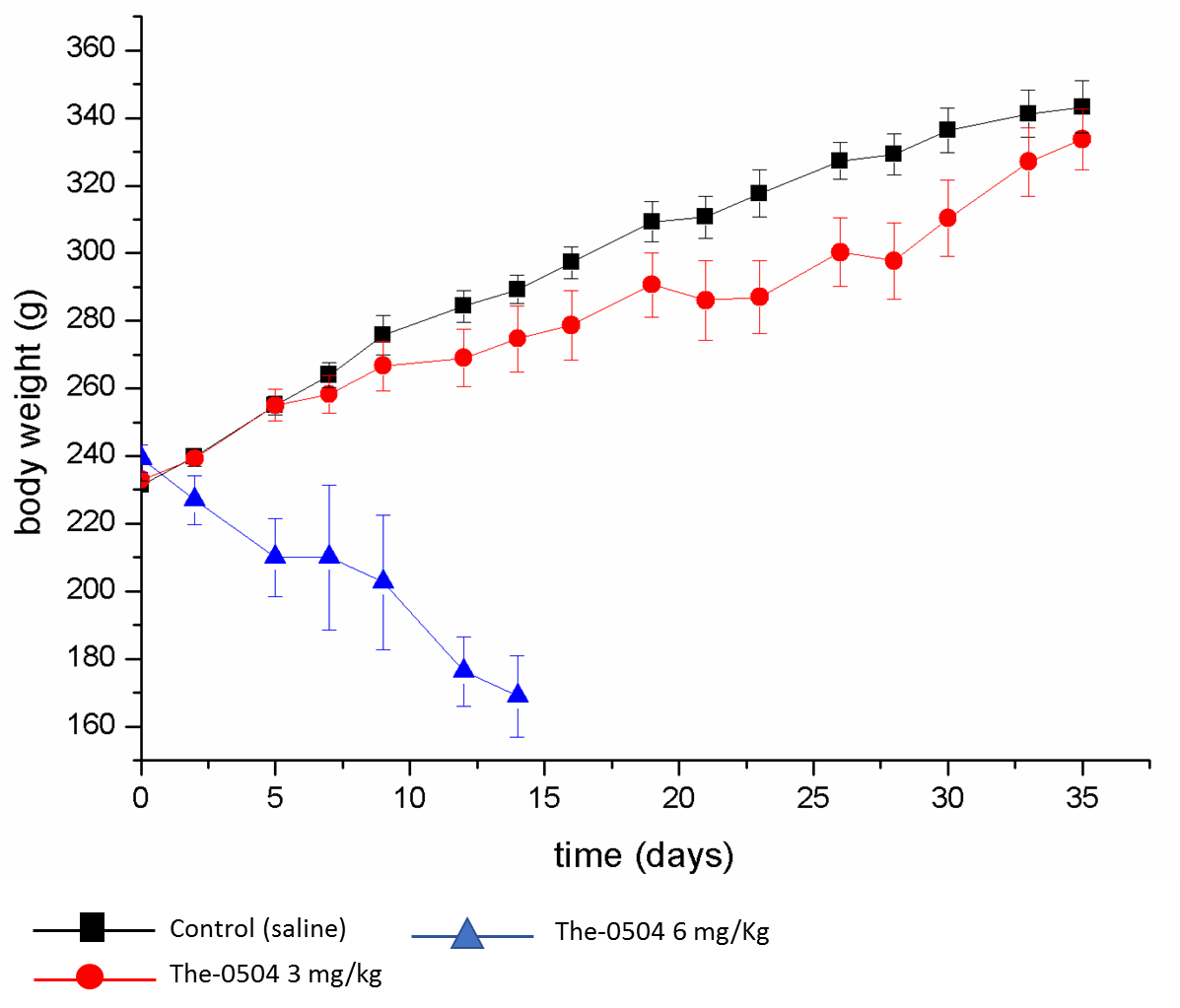


**Fig. S7. Body weight in Wistar rats after The-0504 administration**. The-0504 was injected intravenously once a week for four weeks. Rat body weight was measured three times a week. Control (n=3); The-0504 3 mg/Kg (n=3); The-0504 6 mg/Kg (n=3). All rats of The-0504 6 mg/Kg group were sacrificed after two weeks from the treatments start due to significant body weight loss.


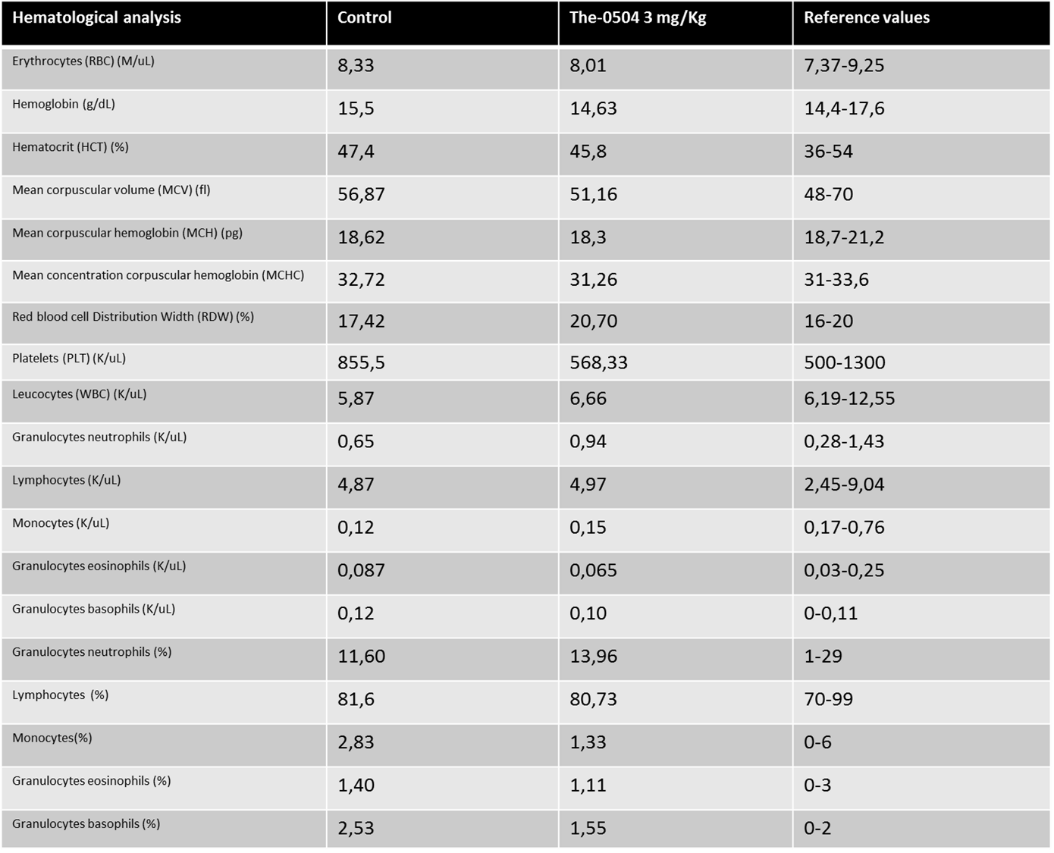


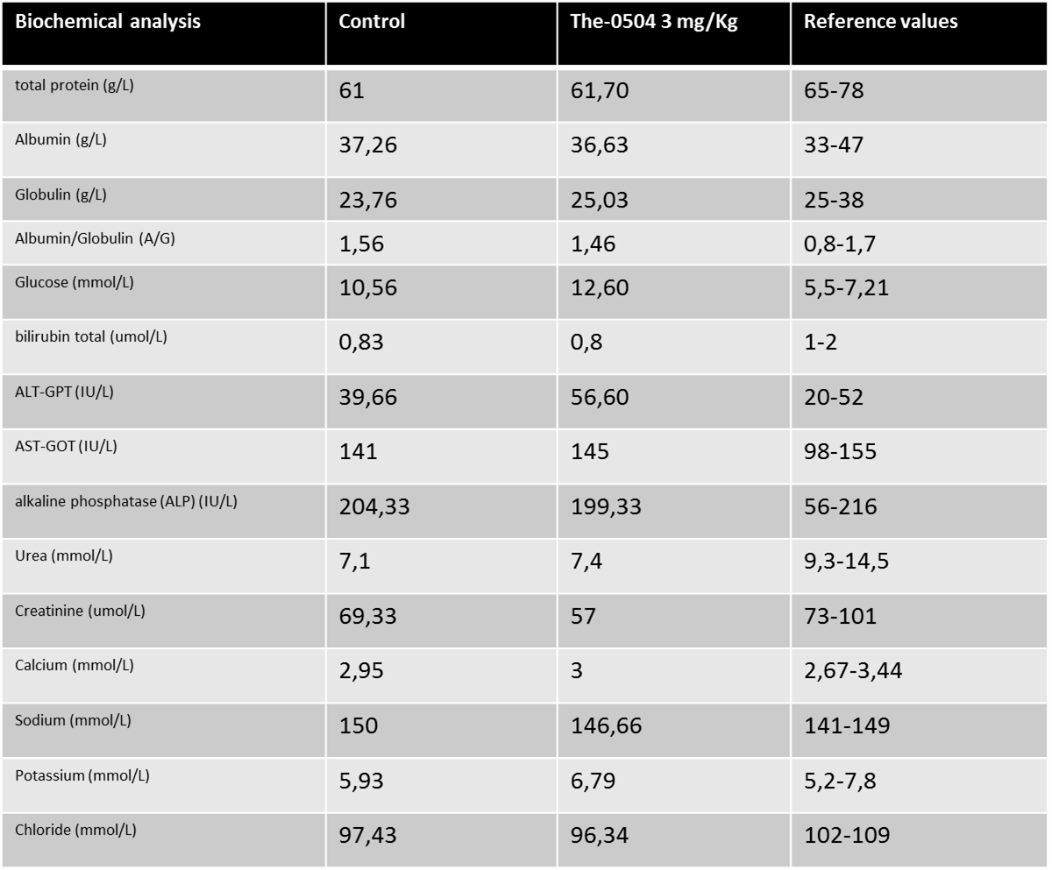


**Fig. S8. Blood sample analysis in Wistar rats after The-0504 administration at 3 mg/Kg**. Hematological (top) and biochemical (bottom) analysis after The-0504 treatment in Wistar rats.


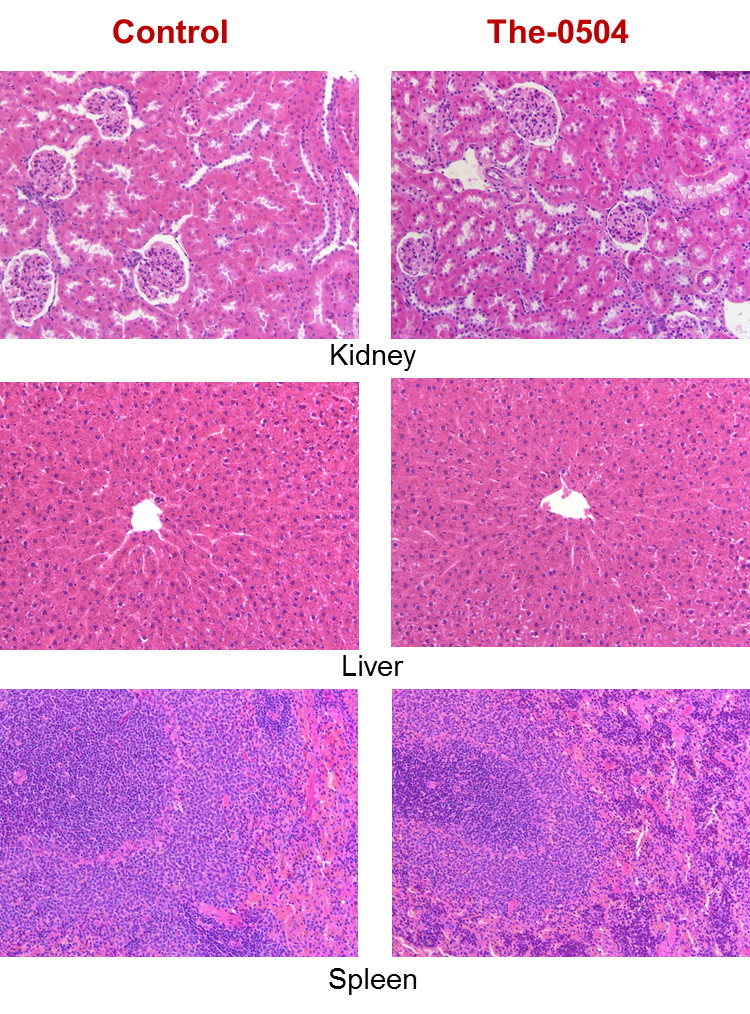


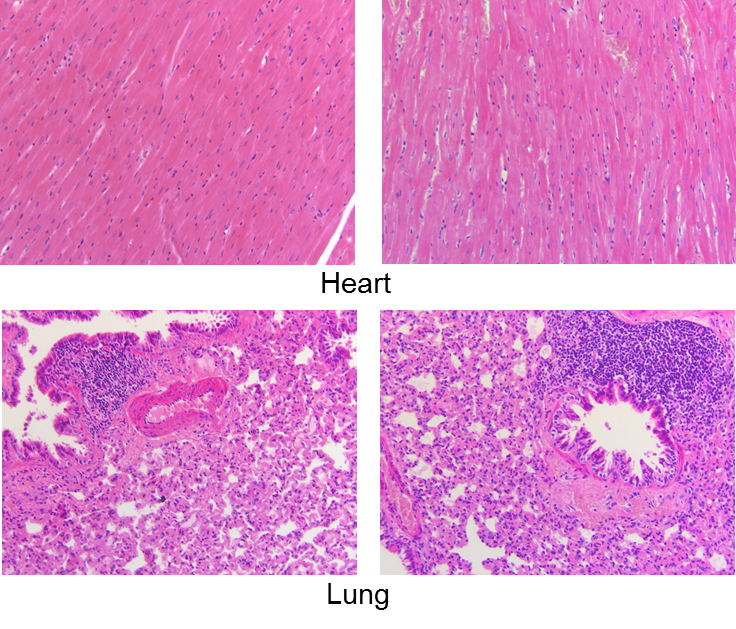


**Fig. S9. Representatives images of immunohistochemistry (IHC).** IHC of control (left) and 3 mg/Kg The-0504 (right) treated rats. Organs are indicated.


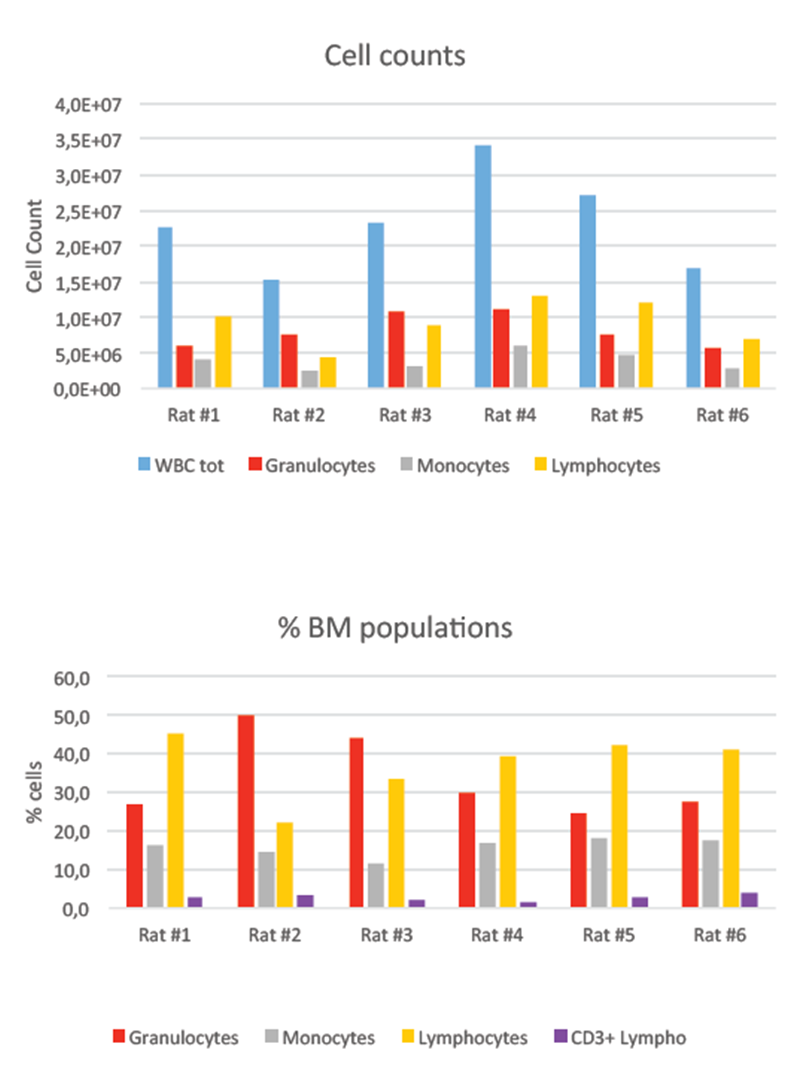


**Fig. S10. Bone marrow cell counts in Wistar rats after The-0504 administration at 3 mg/Kg**. Absolute (top) and relative percentages (bottom) regarding WBC total cells (blue), granulocytes (red), monocytes (gray) and lymphocytes (yellow). Results show a slight increase in WBC population in treated group (rats 4-6) in comparison to control group (rats 1-3) and no differences in the other BM populations.
